# Copy Number Signatures and Clinical Outcomes in Upper Tract Urothelial Carcinoma

**DOI:** 10.3389/fcell.2021.713499

**Published:** 2021-08-26

**Authors:** Bao Guan, Yuan Liang, Huan Lu, Zhengzheng Xu, Yue Shi, Juan Li, Wenwen Kong, Chuanyu Tian, Yezhen Tan, Yanqing Gong, Jin Liu, Dong Fang, Qi Shen, Shiming He, Muhammad Shakeel, Zhongyuan Zhang, Qun He, Xuesong Li, Weimin Ci, Liqun Zhou

**Affiliations:** ^1^Department of Urology, Peking University First Hospital, Beijing, China; ^2^Key Laboratory of Genomics and Precision Medicine, Beijing Institute of Genomics, Chinese Academy of Sciences, Beijing, China; ^3^Institute of Urology, Peking University, Beijing, China; ^4^Beijing Key Laboratory of Urogenital Diseases (Male) Molecular Diagnosis and Treatment Center, National Urological Cancer Center, Beijing, China; ^5^University of Chinese Academy of Sciences, Beijing, China; ^6^Department of Urology, The Third Hospital of Hebei Medical University, Shijiazhuang, China; ^7^Jamil-ur-Rahman Center for Genome Research, PCMD, ICCBS, University of Karachi, Karachi, Pakistan; ^8^Institute of Stem cell and Regeneration, Chinese Academy of Sciences, Beijing, China

**Keywords:** upper tract urothelial carcinoma, whole-genome sequencing, copy number signature, prognosis, cell-free DNA

## Abstract

Tumor staging of upper tract urothelial carcinomas (UTUCs) is relatively difficult to assert accurately before surgery. Here, we used copy number (CN) signatures as a tool to explore their clinical significance of molecular stratification in UTUC. CN signatures were extracted by non-negative matrix factorization from the whole-genome sequencing (WGS) data of 90 Chinese UTUC primary tumor samples. A validation UTUC cohort (*n* = 56) and a cohort from urinary cell-free DNA (cfDNA) of urothelial cancer patients (*n* = 94) and matched primary tumors were also examined. Survival analyses were measured using the Kaplan–Meier, and Cox regression was used for multivariate analysis. Here, we identified six CN signatures (Sig1–6). Patients with a high contribution of Sig6 (Sig6high) were associated with higher microsatellite instability level and papillary architecture and had a favorable outcome. Patients with a low weighted genome integrity index were associated with positive lymph node and showed the worst outcome. Sig6high was identified to be an independently prognostic factor. The predictive significance of CN signature was identified by a validation UTUC cohort. CN signatures retained great concordance between primary tumor and urinary cfDNA. In conclusion, our results reveal that CN signature assessment for risk stratification is feasible and provides a basis for clinical studies that evaluate therapeutic interventions and prognosis.

## Introduction

Recently, the European Association of Urology reported that 90–95% of urothelial carcinoma (UC) occurs as UC of the bladder (UCB), with upper tract UC (UTUC) accounting for 5–10% (Roupret et al., 2018). Clinical interest in UTUC is increasing in East Asian regions, such as China, which have a much higher UTUC prevalence, accounting for more than 30% of UCs (Chen et al., 2012; Hsiao et al., 2016; Yang et al., 2002). Conceivably, geographic differences in risk factors for UTUC, such as aristolochic acid (AA)-containing herb drugs consumption, may account for the observation (Chen et al., 2012; Grollman, 2013; Hoang et al., 2013; Poon et al., 2013). Despite having similar histologic appearance, UTUC is a distinct clinical entity presenting an aggressive clinical behavior and a more advanced presentation compared with UCB (Roupret et al., 2015). However, tumor stage of UTUC is usually difficult to be identified clinically by imageological examination (Roupret et al., 2018). It is useful to “risk stratify” UTUC between low- and high-risk tumors to identify those patients who are more suitable for kidney-sparing surgery or neoadjuvant treatment. However, accurate molecular predictive tools are rare for UTUC, and several available models are mainly based on clinicopathological features (Roupret et al., 2018). Recently, our previous study showed that AA mutational signature defines the low-risk subtype in UTUC (Lu et al., 2020). Additionally, genomic molecular biomarkers, including tumor copy number (CN) alteration (CNA) burden, genomic rearrangement signatures, and mutational signatures, have been reported as independent molecular prognostic makers in different types of cancer (Etemadmoghadam et al., 2009; Hieronymus et al., 2018; Hillman et al., 2018; Staaf et al., 2019). In a word, the genomic variation features may be promising predictive biomarkers for UTUC patients.

Copy number alterations are nearly ubiquitous in cancers (Heitzer et al., 2016; Zack et al., 2013). Recent study showed that tumor CNA burden is a pan-cancer prognostic factor associated with recurrence and death, emphasizing the need to study their biological and clinical significance beyond individual gene-focused standpoints (Hieronymus et al., 2018). Our previous study also showed the genomic heterogeneity of CNA profiles was less prevalent between UTUC and UCB even in different ethnic populations (Ge et al., 2019). Moreover, recent algorithmic advances have enabled interpretation of complex genomic changes by identifying CN signatures with low-depth whole-genome sequencing (WGS) data (Macintyre et al., 2018). It has been shown that CN signatures were associated with clinical outcome in high-grade serous ovarian cancer (Hillman et al., 2018).

Herein, we performed the comprehensive genomic analysis of 90 UTUC patients from a previous study based on CN signatures using WGS approach (Lu et al., 2020). We demonstrated that CN signature could identify genomic subgroups with prognostic significance. Furthermore, the clinical significance of the CN signature subgroups was further validated in another UTUC cohort and UCB patients from The Cancer Genome Atlas (TCGA). Notably, similar CN signatures were also observed in urinary cell-free DNA (cfDNA) of UC patients, suggesting that the CN signature stratification may be applicable in the preoperative setting.

## Materials and Methods

### Patients

**FIGURE 1:**
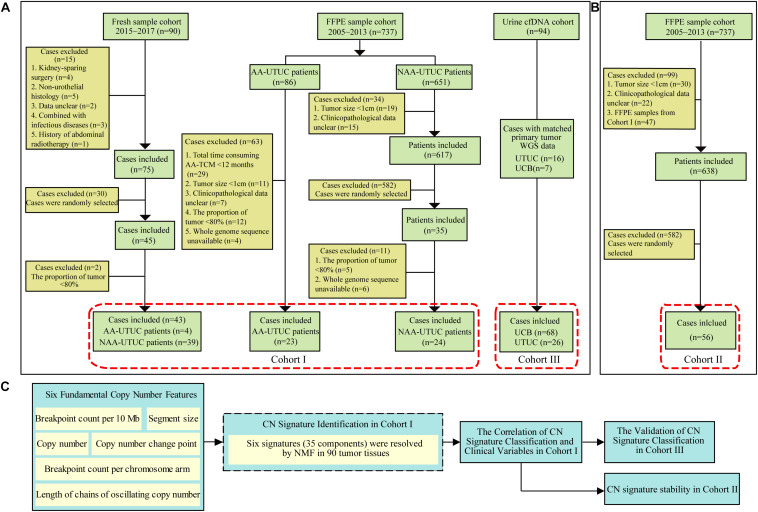
Diagram of patient selection and analysis procedures. **(A)** The WGS data from two cohorts including tumor samples of 90 Chinese UTUC patients (Cohort I) and a validation cohort of 56 UTUC patients (Cohort II). **(B)** Ninety-four preoperative urinary cfDNA of urothelial carcinoma patients (Cohort III: 68 UCB and 26 UTUC). **(C)** All CN signatures identified from Cohort I were used for the extraction of CN signatures from Cohort II and III. WGS, whole-genome sequencing; cfDNA, cell-free DNA; AA, aristolochic acid; NAA, non-aristolochic acid; UTUC, upper tract urothelial carcinoma; FFPE, formalin-fixed paraffin-embedded; AA-TCM, aristolochic acid-associated traditional Chinese medicine; UCB, urothelial carcinoma of bladder; NMF, non-negative matrix factorization; TCGA, The Cancer Genome Atlas; CN, copy number.

Four cohorts of UTUC/UCB were included in this study. In first cohort (Cohort I) (Lu et al., 2020), 43 fresh samples of UTUC were obtained from Peking University First Hospital after ureterectomy or radical nephroureterectomy, between January 2015 and December 2017. And 47 formalin-fixed, paraffin-embedded (FFPE) samples of UTUC were provided by the Institute of Urology after pathologic diagnosis, from January 2005 to December 2013. The fresh samples were snap-frozen in liquid nitrogen immediately after surgery. A total of 56 FFPE samples were randomly selected as a validation cohort (Cohort II). The third independent cohort (Cohort III) consisted of 94 UC patients (including 26 UTUC and 68 UCB patients) with shallow depth (∼5×), for which urine samples were preoperatively collected between June 2016 and December 2018 (Supplementary Table 1; Ge et al., 2020). Matched primary tumor of Cohort III in 16 patients with UTUC and seven patients with UCB was also sequenced (∼5×). The main endpoint events consisted of overall survival (OS), cancer-specific survival (CSS), and metastasis-free survival (MFS). None of the study patients was treated with neoadjuvant treatment. Clinical and demographic information was obtained from a prospectively maintained institutional database. And the last cohort (Cohort IV) composed of TCGA UCB data (*n* = 260) was used for external validation. The flowchart of patient selection is shown in Figures 1A,B. The study was approved by the Ethics Committee of Peking University First Hospital [Grant No. 2018(186)].

### Copy Number Signature Analysis

We sequenced 90 UTUC samples (Cohort I) using WGS (∼30×) for single-nucleotide variation (SNV) and copy number variation (CNV) calling (Baslan et al., 2012; Supplementary Method). To determine CN profiles, we used a published method called Varbin (Baslan et al., 2012). Briefly, the genome was divided into 50,000 bins of variable lengths such that each bin contained normalized equal number of uniquely potential mapping reads. This approach provides advantage over fixed length bins, which creates a bias of non-normalized number of uniquely mapped reads. Furthermore, Circular Binary Segmentation was carried out by DNAcopy (R package), and aCGH (R package) was used to get rid of false-positive breakpoints. We used Integrative Genomics Viewer (IGV) to view the CN profiles (Robinson et al., 2011). The CN signature analysis was then performed according to the protocol described by Macintyre et al. (2018). Concisely, we characterized the CN signatures of the UTUC cohort by six fundamental CN features: the breakpoint count per 10 Mb, the CN of the segments, the difference in CN between adjacent segments, the breakpoint count per chromosome arm, the lengths of oscillating CN segment chains, and the size of segments. Mixture modeling was applied to determine the number of components for each CN feature using FlexMix V2 package of R. Then, non-negative matrix factorization (NMF) (R package) was used to resolve the CN signatures. The samples were allocated to each signature on the basis of the maximal proportion. For clinically associated analysis, samples with weighted genome integrity index (wGII) less than 0.1 were assigned as “wGIIlow” subgroup in this study. Then the remaining samples were halved around the Sig6 contribution into “Sig6high” and “Sig6low” subgroups (cutoff value: ∼0.23). The concise analysis process is shown in Figure 1C. We sequenced the validation cohort (Cohort II) using low-depth WGS (∼3×) for CNV calling, and the CN signature extraction and classification were in terms of the components of 90 UTUC patients (Macintyre et al., 2018).

We calculated the wGII score as reported (Endesfelder et al., 2014). Briefly, the percentage of gained and lost genomic material was calculated relative to the ploidy of the sample. The use of percentages eliminates the bias induced by differing chromosome sizes. The wGII score of a sample was defined as the average of this percentage value over the 22 autosomal chromosomes.

### Copy Number Signatures in an Independent the Cancer Genome Atlas Cohort

Copy number profile segments for each sample in TCGA UCB cohort were filtered to remove segments supported by less than five microarray probes. We deduced each sample’s components and calculated Spearman’s correlation with Sig6 of Cohort I. After one-third low correlation samples (the correlation coefficient lower than ∼0.56) were removed, tumors in TCGA cohort were then bisected around the median Sig6 correlation into “Sig6high” and “Sig6low” subgroups.

### Statistical Analysis

The variables of different groups were compared using the chi-square test, the Wilcoxon rank test, or the Kruskal–Wallis test as indicated. The Kaplan–Meier analysis and Cox proportional hazards model analysis were used to evaluate the associations of the classifiers with OS, CSS, and MFS. The two-sided *p* < 0.05 was considered statistically significant. All analyses were conducted using SPSS version 20.0 (IBM Corporation, Armonk, NY, United States) and R version 3.5.1.

### Data Availability

The raw sequence data reported in this paper have been deposited in the Genome Sequence Archive (Wang et al., 2017) in BIG Data Center (BIG Data Center Members, 2018), Beijing Institute of Genomics (BIG), Chinese Academy of Sciences (accession numbers HRA000029), and can be accessed at http://bigd.big.ac.cn/gsa-human/s/HiObV4f3.

### Data Sources for Integrating Analysis

Genomic sequencing data of bladder tumor specimens were obtained from TCGA data portal^1^. TCGA consortium performed CNA calling and provided level 3 data for CNAs, including segment mean values (*n* = 260). Validation data of 94 UC tumor specimens were obtained from our published WGS data (Ge et al., 2019), which can be accessed at http://bigd.big.ac.cn/gsa-human/s/IjkJWH69.

### Role of the Funding Source

Funding sources did not have any influence on data collection, analysis, or interpretation; trial design; patient recruitment; or any aspect pertinent to the study.

## Results

### Patient Characteristics

Initially, we generated CN profiles from Cohort I using WGS (∼30×). These samples formed the basis of our CN signature identification (Figure 1). The demographic and clinical data of these UTUC patients are shown in Table 1. Herein, the proportion of female patients (61.1%) was slightly higher than that of male patients. Only 17.8% patients had a history of smoking, and 30% patients had consumed AA-containing drugs. The majority of cases had muscle-invasive tumor (52.2%). Metastasis to distant tissues or organs was detected in 31.1% patients during a median follow-up of 31.5 (range 3–168) months. Tumor progression in local and distant organs or in the bladder was detected in 45.6% patients. During the follow-up, 30 patients died, including 28 deaths due to UTUC. The comparison of Cohort I and a validation cohort (Cohort II) is shown in Supplementary Table 2. Cohort II had lower AA intake ratio than had Cohort I (*p* < 0.001).

**TABLE 1:** Clinicopathological characteristics of 90 UTUC patients.

Variable	Number (%)
Total patients	90
Median age at diagnosis, year (IQR)	65(60∼71)
**Gender**	
Female	55(61.1%)
Male	35(38.9%)
**Smoking**	
Absent	74(82.2%)
Present	16(17.8%)
**AA intake**	
Absent	63(70.0%)
Present	27(30.0%)
**Synchronous bladder cancer**	
Absent	85(94.4%)
Present	5(5.6%)
**History of bladder cancer**	
Absent	81(90.0%)
Present	9(10.0%)
**CKD**	
1	9(10.0%)
2	29(32.2%)
3	38(42.2%)
4	2(2.2%)
5	12(13.2%)
**Location**	
Pelvis	58(64.4%)
Ureter	32(35.6%)
**Multifocality**	
Absent	78(86.7%)
Present	12(13.3%)
**Size**	
<3 cm	38(42.2%)
≥3 cm	52(57.8%)
**Architecture**	
Papillary	64(71.1%)
Sessile	26(28.9%)
**T stage**	
Ta	1(1.1%)
T1	42(46.7%)
T2	25(27.8%)
T3	20(22.2%)
T4	2(2.2%)
**Grade**	
Low	25(27.8%)
High	65(72.2%)
**N stage**	
N0 or Nx	83(92.2%)
N1	5(7.8%)
N2	2(2.2%)
Postoperative chemotherapy	9(10.0%)
Postoperative radiotherapy	5(5.6%)
**Survival data**	
Cancer-related death	28(31.1%)
Overall death	30(33.3%)
Metastasis	28(31.1%)
Bladder recurrence	19(21.1%)
Cancer progression	41(45.6%)
Median FU (IQR)	31.5(24∼84.75)
Median FU for surviving patients, month (IQR)	32(24.75∼86.25)

*UTUC, upper tract urothelial carcinoma; IQR, interquartile range; AA, aristolochic acid; CKD, chronic kidney disease; FU, follow-up.*

### Copy Number Alterations in 90 Upper Tract Urothelial Carcinoma Patients Defined Six Copy Number Signatures

**FIGURE 2:**
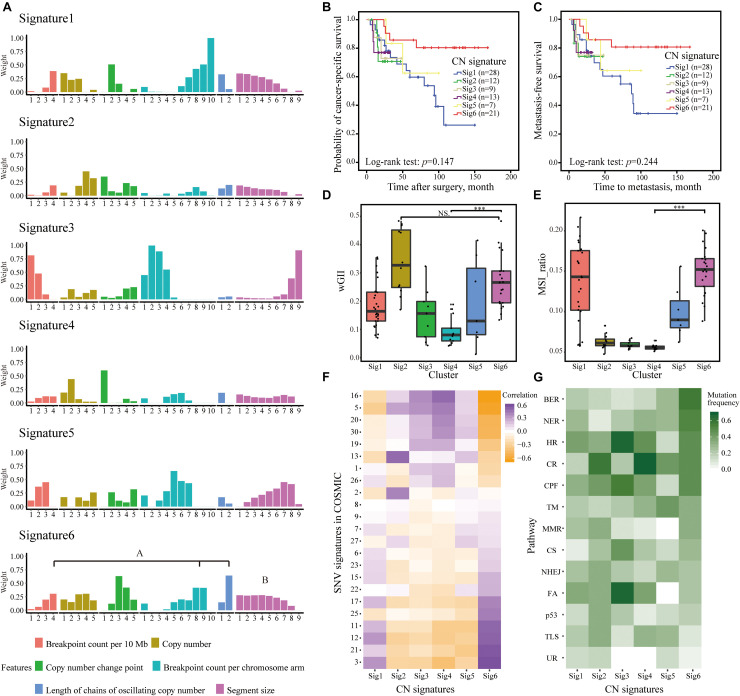
CNAs in 90 UTUC patients defined six CN signatures. **(A)** The component weights for six CN signatures. The important components weights including high numbers of breakpoints, long chains of oscillating copy numbers, and fragmented genomic segments of Sig6 were highlighted. **(B,C)** The Kaplan–Meier analysis of cancer-specific survival **(B)** and metastasis-free survival **(C)** in the UTUC Cohort I stratified by six CN signatures. Statistical significance was determined by log-rank test. **(D,E)** Comparison of wGII **(D)** and MSI **(E)** among different CN signature exposures derived from WGS data. Statistical significance was determined by Wilcoxon rank test, ***p < 0.001. NS represents not significant. **(F)** Associations between CN signature exposures and SNV signatures in COSMIC. **(G)** Difference among CN signature exposures in mutations of genes involving in multiple DNA damage response pathways. AM, alternative mechanism for telomere maintenance; BER, base excision repair; CPF, checkpoint factor; CNA, copy number alteration; CR, chromatin remodeling; CS, chromosome segregation; DR, direct repair; FA, Fanconi anemia pathway; HR, homologous recombination; MMR, mismatch repair; NER, nucleotide excision repair; NHEJ, non-homologous end joining; TLS, translesion synthesis; TM, telomere maintenance; UR, ubiquitination response; CN, copy number; wGII, weighted genome integrity index score; MSI, microsatellite instability; WGS, whole-genome sequencing; SNV, single-nucleotide variation; UTUC, upper tract urothelial carcinoma.

We identified six CN signatures, comprising six features of 35 components in Cohort I (Figure 2A). The majority of our cases exhibited multiple signature exposures, suggesting that UTUC genomes are shaped by more than one mutational process (Supplementary Figure 1). After allocating samples according to the maximum proportion of the signatures, we examined the association of the CN signatures classification and clinical outcome. The Kaplan–Meier plots showed that patients with Sig6 exhibited favorable clinical outcome (Figures 2B,C). For Sig6, the highest weights were observed for components that represent high numbers of breakpoints, long chains of oscillating CNs, and fragmented genomic segments (Figure 2A), suggesting higher level of genomic instability. In contrast, least weightage of contributing components and slightest CN changes was observed in Sig4 (Figure 2A), implying lower level of genomic instability. Owing to this hypothesis, the wGII level and microsatellite instability (MSI) were determined for CN signatures. Consistently, Sig4 was correlated with the lowest wGII level and MSI, but Sig6 was the opposite (Figures 2D,E). Moreover, we correlated CN signatures with COSMIC SNV signatures derived from WGS point mutational data (Tate et al., 2019). Strikingly, Sig6 was positively correlated with SNV signatures 3, 21, 12, and 11 and negatively correlated with SNV signatures 16, 5, 20, and 30, which was also in stark contrast to Sig4 (Figure 2F). SNV signature 3 is associated with failure of DNA double-strand break-repair by homologous recombination, SNV signature 21 is correlated with microsatellite unstable tumors, and SNV signature 11 is correlated with deficiently in transcription-coupled nucleotide excision repair (Tate et al., 2019). Notably, Sig6 was enriched in patients with higher frequent mutations in genes of multiple DNA damage pathways, such as base excision repair, nucleotide excision repair, and homologous recombination (Figures 2F,G). Taken together, genomic instability might be the underlying mechanism for Sig6.

### Copy Number Signature Subgroups and Clinical Outcomes

**FIGURE 3:**
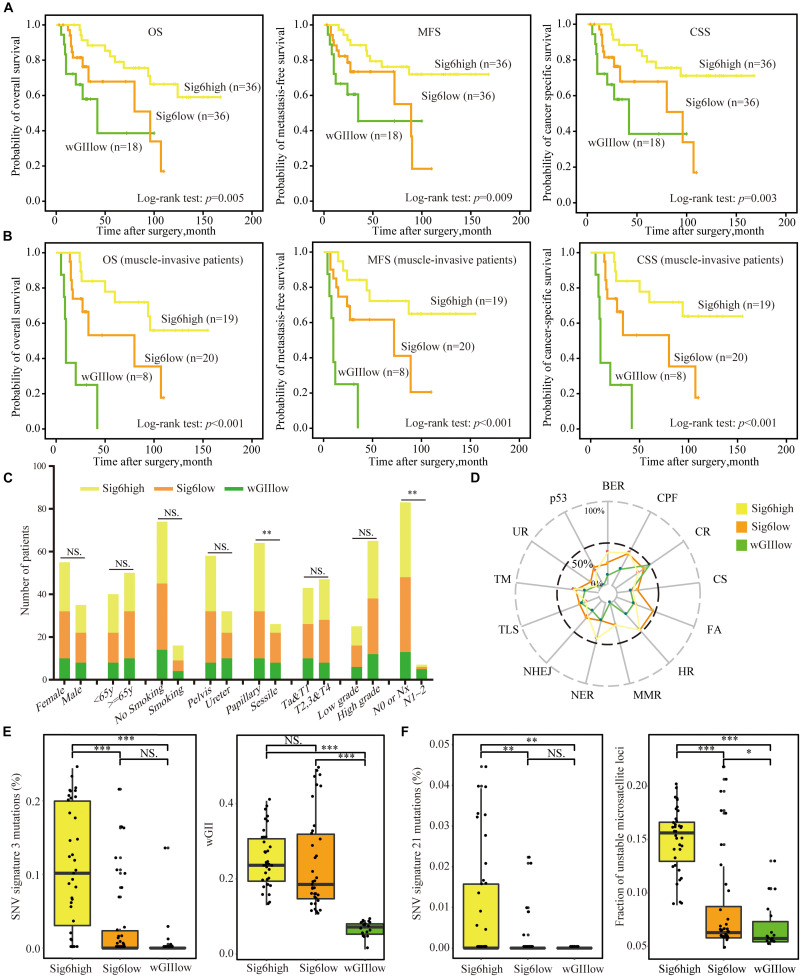
The association of copy number CN signature and clinical features. **(A,B)** The Kaplan–Meier analysis of overall survival, metastasis-free survival, and cancer-specific survival in Cohort I **(A)** and in muscle-invasive tumor patients **(B)**. Statistical significance was determined by log-rank test. **(C)** The bar graph shows the association between the three CN signature subgroups and clinicopathological features. Statistical significance was determined by Kruskal–Wallis test, ^∗∗^*p* < 0.01; and NS indicates not significant. **(D)** The proportion of samples mutated in DNA damage repair pathways of the three CN signature subgroups. **(E)** CN Sig6high subgroup patients had higher number of SNV signature 3 mutations and higher level of wGII than had wGIIlow subgroup patients. Statistical significance was determined by Wilcoxon rank test, ^∗∗∗^*p* < 0.001; NS indicates not significant. **(F)** Patients with Sig6high subgroup had higher number of SNV signature 21 mutations and higher level of MSI than had wGIIlow subgroup patients. Statistical significance was determined by Wilcoxon rank test, ^∗^*p* < 0.05, ^∗∗^*p* < 0.01, ^∗∗∗^*p* < 0.001; and NS represents not significant. CN, copy number; wGII, weighted genome integrity index score; MSI, microsatellite instability; WGS, whole-genome sequencing; SNV, single-nucleotide variation; UTUC, upper tract urothelial carcinoma; AM, alternative mechanism for telomere maintenance; BER, base excision repair; CPF, checkpoint factor; CR, chromatin remodeling; CS, chromosome segregation; DR, direct repair; FA, Fanconi anemia pathway; HR, homologous recombination; MMR, mismatch repair; NER, nucleotide excision repair; NHEJ, non-homologous end joining; TLS, translesion synthesis; TM, telomere maintenance; UR, ubiquitination response.

Since patients with Sig6 tend to have better clinical outcomes (Figure 2B), we divided Cohort I into three subgroups based on the proportion of Sig6: (1) Sig6high, which possessed higher proportion of Sig6; (2) Sig6low, which possessed lower proportion of Sig6; and (3) wGIIlow (wGII less than 0.1), which did not exhibit a dominant CN feature (Supplementary Figure 2 and Supplementary Table 3). The Kaplan–Meier plots demonstrated that the Sig6high subgroup showed favorable OS, MFS, and CSS, but the wGIIlow subgroup showed poor clinical outcomes (Figure 3A, *p* < 0.01). Moreover, the Sig6high subgroup showed the best clinical outcomes in patients with muscle-invasive tumor (Figure 3B, *p* < 0.01). With the use of multivariate Cox regression proportional analyses adjusted for the effects of clinicopathological variables, the contribution of Sig6 was an independent prognostic factor for CSS [hazard ratio (HR) = 3.207, 95% CI: 1.316∼7.814, *p* = 0.01] and MFS [HR = 2.606, 95% CI: 1.084∼6.265, *p* = 0.032] (Table 2).

**TABLE 2:** Univariate and multivariate Cox regression analyses predicting cancer-specific survival and metastasis-free survival.

Variables	Cancer specific survival						Metastasis-free survival					
	Univariate			Multivariate			Univariate			Multivariate		
	HR	95% CI	*P*	HR	95% CI	*p*	HR	95% CI	*p*	HR	95% CI	*p*
Age (<65 vs. ≥65 years)	1.576	0.725∼3.427	0.251				1.618	0.743∼3.525	0.226			
AA intake (present vs. absent)	0.283	0.111∼0.724	**0.008**	0.297	0.105∼0.839	**0.022**	0.307	0.120∼0.786	**0.014**			
Gender (female vs. male)	0.837	0.364∼1.924	0.676		∼		0.769	0.336∼1.762	0.535			
Location (ureter vs. pelvis)	2.574	1.214∼5.459	**0.014**		∼		2.592	1.218∼5.517	**0.013**	2.290	1.038∼5.048	**0.040**
Multifocality (absent vs. present)	0.597	0.180∼1.980	0.399		∼		0.623	0.188∼2.067	0.439			
Tumor size (<3 vs. ≥ 3 cm)	0.847	0.402∼1.782	0.662		∼		0.810	0.385∼1.705	0.580			
Architecture (papillary vs. sessile)	1.546	0.673∼3.552	0.304		∼		1.412	0.618∼3.230	0.413			
T stage (T2, T3, and T4 vs. Ta, 1)	3.632	1.472∼8.961	**0.005**	3.563	1.426∼8.900	**0.007**	3.687	1.495∼9.098	**0.005**	3.561	1.437∼8.821	**0.006**
Grade (low vs. high)	2.580	0.895∼7.442	0.079		∼		2.629	0.911∼7.584	0.074			
N (N1∼2 vs. N0/Nx)	5.733	2.106∼15.609	**0.001**	3.024	1.080∼8.464	**0.010**	5.627	2.069∼15.300	**0.001**	4.558	1.631∼12.742	**0.004**
CNV cluster (Sig6high vs. Sig6low and wGIIlow)	3.506	1.513∼8.127	**0.003**	3.207	1.316∼7.814	**0.010**	3.120	1.347∼7.231	**0.008**	2.606	1.084∼6.265	**0.032**
MSI_ratio (≥0.15 vs. <0.15)	0.366	0.149∼0.896	**0.028**				0.398	0.162∼0.979	**0.045**			

*Nx, no lymph node dissection was performed. P-value smaller than 0.01 is indicated in bold.*

Due to the high proportion of AA exposure brought by FFPE sample selection bias in Cohort I (Supplementary Table 1), we verified the correlation of CN signature classification and clinical outcomes in the patients of no-AA Sig subgroup, which showed weak AA mutational signature (Lu et al., 2020). The Kaplan–Meier plots showed the CN signature classification was related to CSS, OS, and MFS, especially in muscle-invasive tumor patients (Supplementary Figure 3), and multivariate Cox regression analyses found that the CN signature classification did independently predict outcome for no-AA Sig subgroup patients (Supplementary Table 4). It is implied that this predictive model based on CN signature probably was not influenced by AA exposure.

The chi-square test showed that the Sig6high subgroup was related to AA intake and papillary tumor architecture, but the wGIIlow subgroup showed a positive correlation with positive lymph node (Figure 3C and Supplementary Table 5). Besides, the wGIIlow subgroup had the lowest number of both stromal tumor-infiltrating myeloid cells (TIMCs) and CD3^+^ lymphocytes (Supplementary Figure 4, *p* < 0.05), which suggested that the wGIIlow subgroup had poor prognosis (Lipponen et al., 1992).

Moreover, we explored the association of SNV signatures derived from WGS point mutations with the Sig6-based subgroups. The wGIIlow subgroup showed higher mutational frequency in chromatin remodeling DNA damage pathway (Figure 3D). The genes of this pathway have been reported to be frequently mutated in advanced bladder tumors (Gui et al., 2011). The Sig6high group showed higher mutations in SNV signatures 3 and 21 and higher MSI level, which is an independent molecular prognostic maker for UTUC (Roupret et al., 2005), than did the other two subgroups. Sig6high and Sig6low subgroups were associated with higher wGII ratio than the wGIIlow subgroup (Figures 3E,F, *p* < 0.05). In addition, the IGV figure showed that the patients in Sig6low subgroup had more CNVs than the wGIIlow subgroups but had larger segments of CNVs than the Sig6high subgroup (Supplementary Figure 2).

### Validation of Prognostic Copy Number Signature and Non-Invasive Monitoring of Copy Number Signatures

**FIGURE 4:**
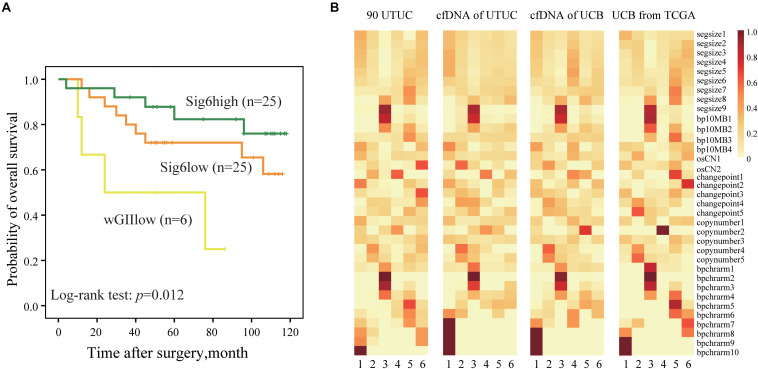
The predictive significance of copy number CN signature. **(A)** The Kaplan–Meier analysis of overall survival in a UTUC validation cohort. **(B)** Heatmaps show component weights of CN signatures in Cohort I, Cohort III, and Cohort VI. UTUC, upper tract urothelial carcinoma; cfDNA, cell-free DNA; UCB, urothelial carcinoma of bladder; CN, copy number.

A validation cohort including 56 randomly selected UTUC patients (Cohort II) was used to validate the predictive value of CN signature classification. Consistent to the result of Cohort I, we found that the Sig6high subtype had a better OS than the wGIIlow subtype (Figure 4A, *p* = 0.012). Interestingly, we also evaluated the association between Sig6-based subgroup classification and prognosis using TCGA UCB data. After removing the samples with lower Spearman’s correlation with Sig6 and unclear clinical prognostic information, we divided 260 TCGA UCB samples into Sig6high and Sig6low subgroups. The Kaplan–Meier plots showed that the Sig6high group also showed better OS and MFS than the Sig6low subgroup (Supplementary Figure 5, *p* < 0.05).

Our previous study showed that genome-wide CN profiles in urinary cfDNA are highly concordant with primary tumor specimens in both UTUC and UCB (Ge et al., 2019). Thus, we explored whether CN signatures can also be assessed non-invasively using urinary cfDNA. Spearman’s correlation showed that CN signatures in cfDNA had higher concordance with primary tumor specimens compared with CN profiles in both UTUC and UCB patients (Supplementary Figures 6A,B), which may infer that CN signatures may be a more stable marker than CN profile. Moreover, by comparing CN signatures patterns derived from UCB patients from TCGA (Cohort IV), urinary cfDNA patients (Cohort III), and UTUC patients (Cohort I), similar CN signatures patterns were observed (Figure 4B). However, due to limited follow-up data in urinary cohort, we could not further evaluate the prognostic significance of CN signatures in patients with UTUC.

## Discussion

In this study, we applied the recently developed genomic methods (Macintyre et al., 2018) to explore prognostic CN signatures and mechanisms underlying the CN components in UTUC patients. We found tumors with high contributions of Sig6 independently related to a favorable outcome. Moreover, Sig6 was less positively associated with SNV signature 22, the AA-associated mutational signature, and a typical hallmark of UTUC genome in East Asians (Figure 2F). Consistently, similar CN profiles and CN signatures were derived from AA and no-AA cohort of patients (Lu et al., 2020; Supplementary Figure 7). These results indicated that CN signatures may remain consistent in patients with UTUC irrespective of prior AA exposure. Furthermore, we provided a proof of concept that the CN signatures remain consistent between UTUC and UCB. Thus, we also validated the predictive effects of Sig6 in the independent UTUC cohort and TCGA UCB cohort. Additionally, several studies had verified that WGS is a robust method for obtaining good-quality CNA data from FFPE cancer samples (Chin et al., 2018; Kader et al., 2016; Scheinin et al., 2014). We also compared the CN signature components between fresh samples and FFPE samples, and very similar patterns were observed (Supplementary Figure 8). Moreover, we can also derive highly consistent CN signatures using inexpensive shallow WGS of both core biopsies and liquid biopsies. These approaches are rapid and cost-effective, thus providing a practical path to clinical implementation.

The prognostic significance of CN signatures raises intriguing questions regarding the underlying biology. Tumor CN signature may be a simple measure that correlates with the extent of oncogenic driver alterations, such as wGII and MSI level. Yet we showed that tumor CN signature retains its prognostic significance after adjustment for clinicopathological information and genomic alterations in UTUC (Table 2). Moreover, the Sig6high subgroup was featured by SNV signature 3 in COSMIC, which is exhibited in responders to platinum therapy in pancreatic cancer (Greer and Whitcomb, 2007). These results provided basis to prospective clinical studies of platinum-based chemotherapy in UTUC patients belonging to CN Sig6high subgroup. Additionally, higher frequency mutations in genes of multiple DNA damage repair (DDR) pathways were identified in the Sig6high subgroup of patients. Classically, defects in the DDR pathways have been exploited therapeutically in the treatment of cancer with radiation therapies or genotoxic chemotherapies. Further mechanistic insights of the CN signature subgroups may provide the basis for therapeutic opportunities within the DNA damage response. However, in current Cohort I, none of the patients was treated with neoadjuvant chemotherapy, and after radical nephroureterectomy for the advance diseases, very few patients can receive chemotherapy because of comorbidity and impaired renal function. Thus, we cannot evaluate the therapeutic relevance of Sig6 high-similarity subgroup in our cohort. The consistency of urine cfDNA with the solid tumor suggests that the CN signature-related subtype of the tumor can be obtained non-invasively using the cfDNA, which can provide better suggestions for risk assessment of potential treatment therapy and prognosis evaluation. For a patient with ureter cancer diagnosed with the Sig6high subtype from urine cfDNA, a kidney-sparing surgery would be more appropriate when there is no radiological evidence for advanced primary tumor or lymph node metastasis. And when the lesion progresses to non-organ localized tumor, neoadjuvant and adjuvant chemotherapies, and immune checkpoint inhibitor would be promising treatments due to the association of the Sig6high subtype and DDR pathway and high immune-associated cell infiltration. Prospective validation of the prognostic value and therapeutic relevance of Sig6 would result in a clinically applicable biomarker for UTUC and UCB. Such biomarker could be used to identify patients with a favorable prognosis who may be candidates for clinical trials in the adjuvant setting.

In this study, we found that the wGIIlow subgroup was associated with low tumor-infiltrating lymphocytes, which were reported as an independently prognostic factor in bladder cancer (Lipponen et al., 1992), and positive lymph node, a well-known predictive marker. These results demonstrated that a lower level of genome instability was an unfavorable genomic molecular factor for UTUC patients (Figure 3, *p* < 0.05). It was also shown that patients in the wGIIlow subgroup should be considered to perform a systemic treatment regimen and undergo preventative lymph node dissection. In addition, because CN signature classification was related to lymph node metastasis and prognosis status, and not related to tumor stage and grade, it may indicate that the CN signatures reflect the invasion and migration capacity of tumor instead of tumor proliferation. However, the mechanism of low genome instability leading to poor prognosis remains to be studied.

One of the limitations of this work is the retrospective nature, which we cannot eliminate the selection bias from single-center data. Besides, a multicenter study with a large scale of cohort is needed to validate the predictive value of CN signature classification. Meanwhile, a large number of paired samples and long-term follow up are required to further evaluate the robustness and effectiveness of non-invasive detection from urine.

In summary, CN signatures showed a close correlation to the relevant mutational processes, and the CN signature subgroup assessment for risk stratification is feasible in UTUC. CN signatures also revealed the potentiality in clinical application like non-invasive monitoring of clinical outcome and provided a basis for prospective clinical studies that evaluate therapeutic interventions.

## Data Availability Statement

The data presented in the study are deposited in the Genome Sequence Archive in BIG Data Center, Beijing Institute of Genomics (BIG), Chinese Academy of Sciences, accession number HRA000029.

## Ethics Statement

The studies involving human participants were reviewed and approved by the Ethics Committee of Peking University First Hospital. The patients/participants provided their written informed consent to participate in this study.

## Author Contributions

WC supervised all the studies. LZ, WC, and XL designed the study. BG, HL, YS, JL, WK, and YT performed the acquisition of the data and sequencing. HL, BG, and YL analyzed and interpreted the data. HL, YL, ZX, and CT performed the statistical analysis. HL, BG, YL, and WC drafted the manuscript. HL, BG, YL, MS, and WC revised the manuscript. YG, JL, and DF contributed to the sample collection and clinical information. QS contributed to material support. SH, ZZ, and QH contributed to administrative and technical support. All authors read and approved the final version of the manuscript.

## Conflict of Interest

The authors declare that the research was conducted in the absence of any commercial or financial relationships that could be construed as a potential conflict of interest.

## Publisher’s Note

All claims expressed in this article are solely those of the authors and do not necessarily represent those of their affiliated organizations, or those of the publisher, the editors and the reviewers. Any product that may be evaluated in this article, or claim that may be made by its manufacturer, is not guaranteed or endorsed by the publisher.

## Footnotes

1
https://portal.gdc.cancer.gov/
