# Supplementary material for: Copy Number Signatures and Clinical Outcomes in Upper Tract Urothelial Carcinoma
Source: Front Cell Dev Biol. 2021 Aug 26;9:713499. doi: 10.3389/fcell.2021.713499 (PMC8427613; doi:10.3389/fcell.2021.713499)
Supplement: Supplementary file 1 [file Data_Sheet_1.docx]

**Supplementary Materials:**

**Supplementary Methods**

***Whole-genome sequencing***

For whole-genome sequencing, genomic DNA from FFPE cancer samples was isolated using the Quick-DNA™ FFPE Kit and Genomic DNA Clean & Concentrator (Zymo Research, CA, US) and from fresh cancer tissue samples using the QIAmp DNA Mini Kit (QIAGEN Inc., MD, US). The sheared DNA was repaired and 3’ dA-tailed using the NEBNext Ultra II End Repair/dA-Tailing Module unit and then ligated to paired-end (PE) adaptors using the NEBNext Ultra II Ligation Module unit. After purification by AMPure XP beads, the DNA fragments were amplified by PCR for 6-8 cycles. The urinary cfDNA first removed large DNA fragments by 0.6X AMPure XP beads for size selection. Then we enriched the fragments with a range of 100 to 300-bp using 0.3X beads. After that the DNA was end repaired, dA tailed, and amplified by PCR for 8-11 cycles. The detailed information of cfDNA has been reported by Ge et al. [[1](#_ENREF_1)]. Finally, the libraries were sequenced with an Illumina HiSeq X10 instrument, thus generating 2 x 150-bp PE reads. The single nucleotide variations calling had been described by Lu et al. [[2](#_ENREF_2)].

***SNV calling and COSMIC SNV signatures identification***

We calculated the wGII score The SNV calling and COSMIC SNV signatures was performed as our previous study [[3](#_ENREF_3)]. Next, we explored the dynamic interplay of risk factors and cellular processes using mutational signature analysis. We identified 23 mutational signatures defined by COSMIC in our cohort by MutationalPatterns [[4](#_ENREF_4)]. The correlation analysis was performed based on previous report [[5](#_ENREF_5)]. Cor function (R package) was used to calculate the correlation between SNV signatures and CN signatures.

***Microsatellite instability (MSI) analysis***

For MSI analysis, mSINGS [[6](#_ENREF_6)] was used to calculate the ratio of unstable loci in the genome of each sample. First, the microsatellites at the reference genome were calculated by MSIsensor [[7](#_ENREF_7)]. Sixteen microsatellite stable esophageal squamous cell carcinomas [[8](#_ENREF_8)] were used to calculate the baseline for the absence of MSI signature (signature 6, 15, 20, 21, 26, 27) as defined by COSMIC [[9](#_ENREF_9)].

***Assessing tumor-infiltrating lymphocytes***

The tumor infiltrating mononuclear lymphocytes were measured according to a standardized method from the International Immuno-Oncology Biomarkers Working Group [[10](#_ENREF_10)]. The CD3 antibody (ab5690, 1:10000; Abcam) was used to evaluate the CD3^+^ lymphocytes in tumor section.

**Supplementary Figures 1-8:**

**
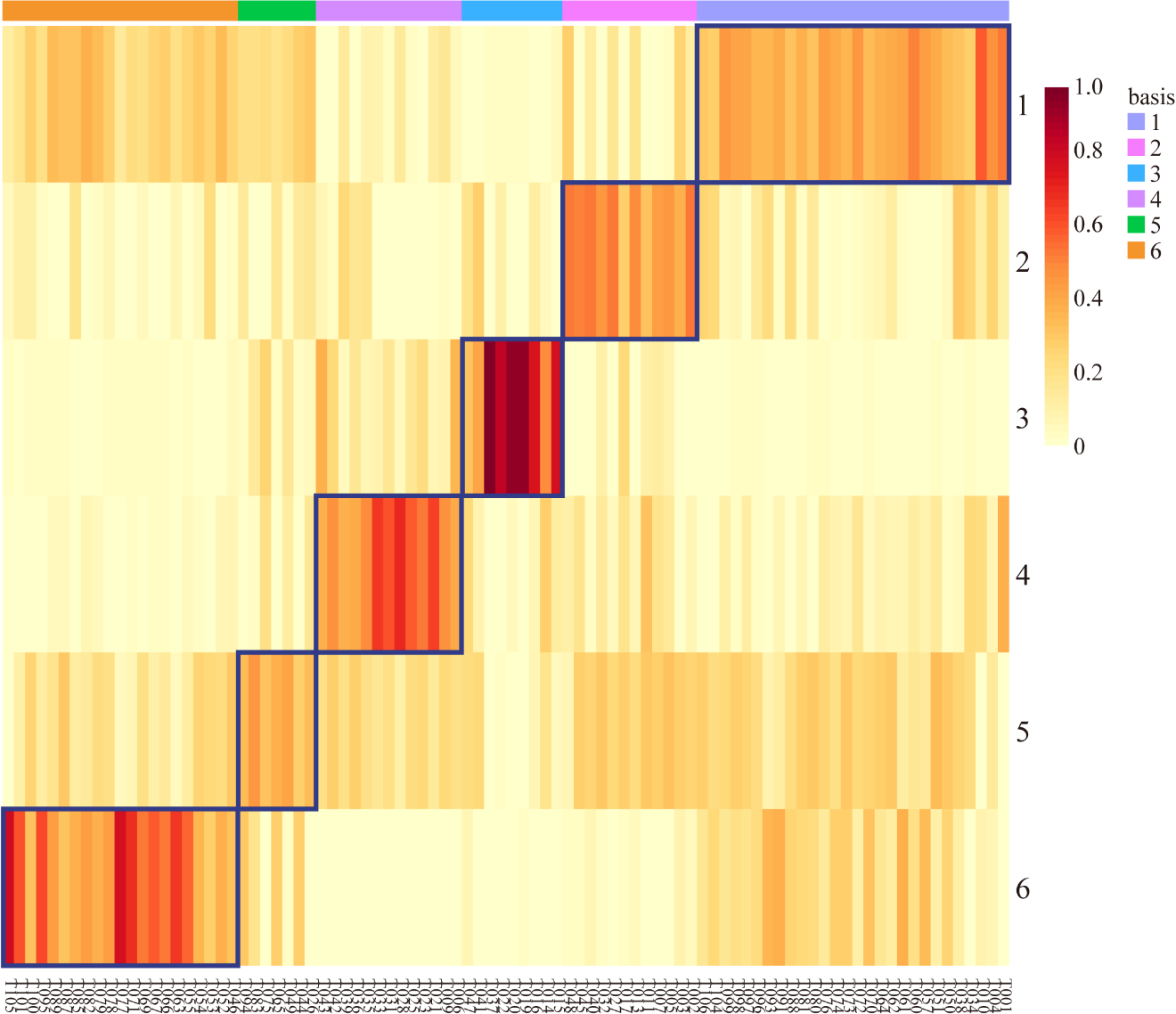
**

**Supplementary Figure 1.** Patient-by-signature matrix of 90 UTUC samples. Patient-by-signature matrix derived by NMF showed that UTUC patients exhibited multiple signature exposures.

**
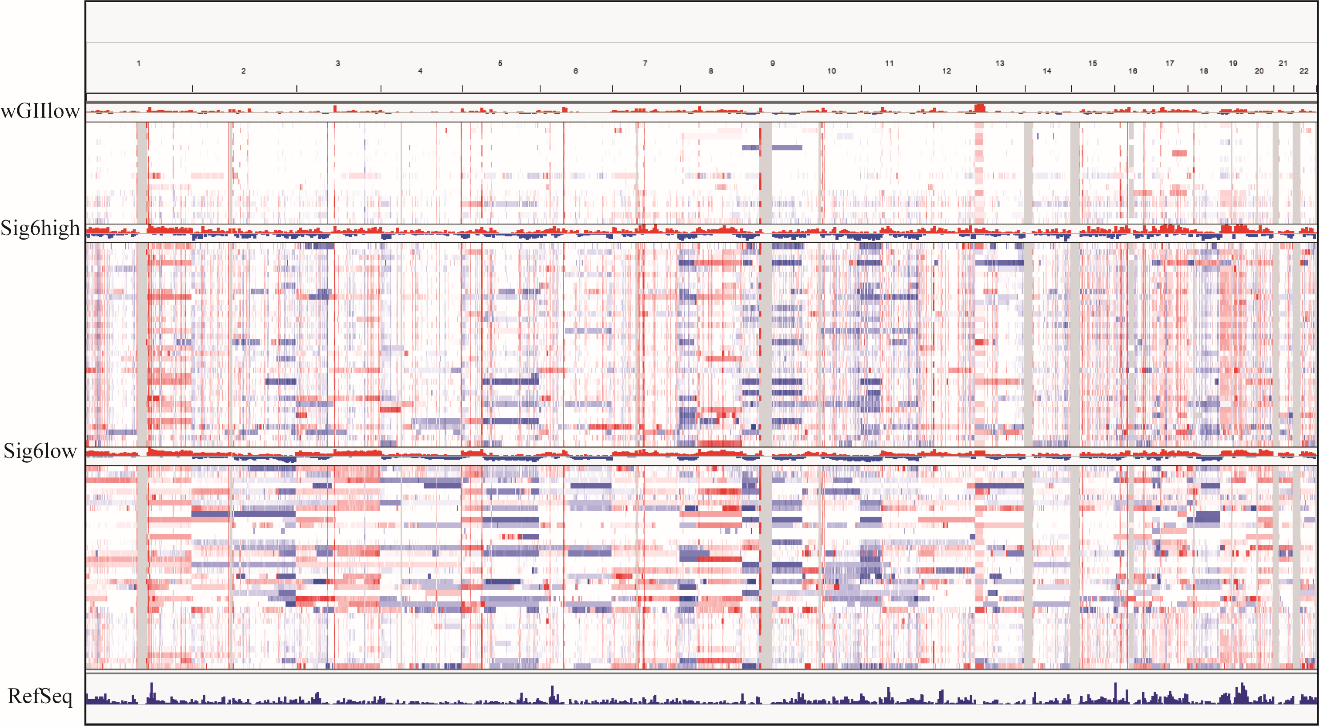
**

**Supplementary Figure 2.** CNA patterns of three subgroups using IGV. wGIIlow subgroup (top) has the least CNAs. Sig6high subgroup (middle) showed more fragmented CNAs than Sig6low subgroup (below).


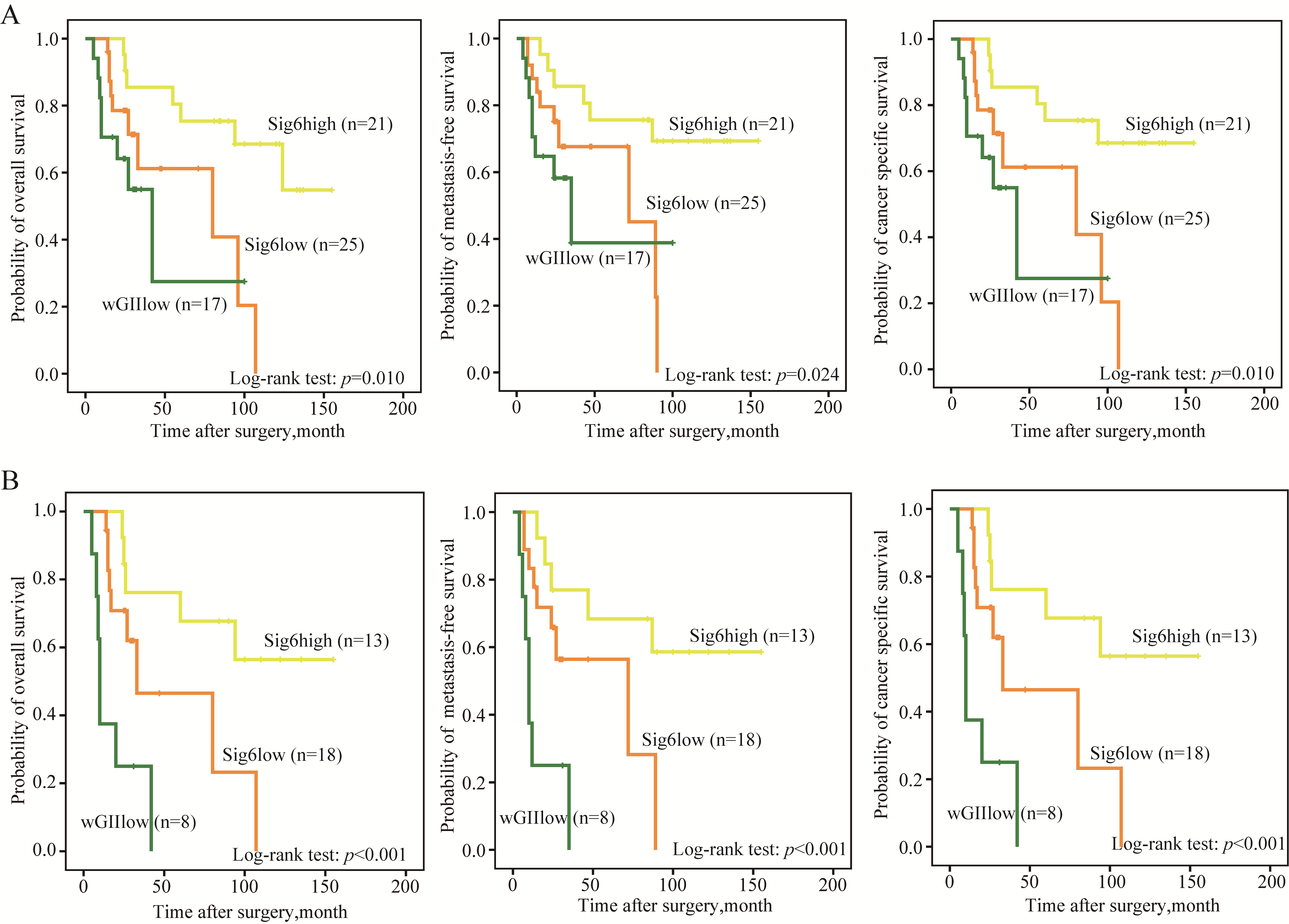


**Supplementary Figure 3.** The association of CN signature and clinical features in patients of no-AA Sig subgroup*.* **(A, B)** Kaplan-Meier analysis of overall survival (left), metastasis-free survival (middle) and cancer-specific survival (right) in 63 patients of no-AA Sig subgroup **(A)** and in 39 muscle-invasive patients **(B)**.


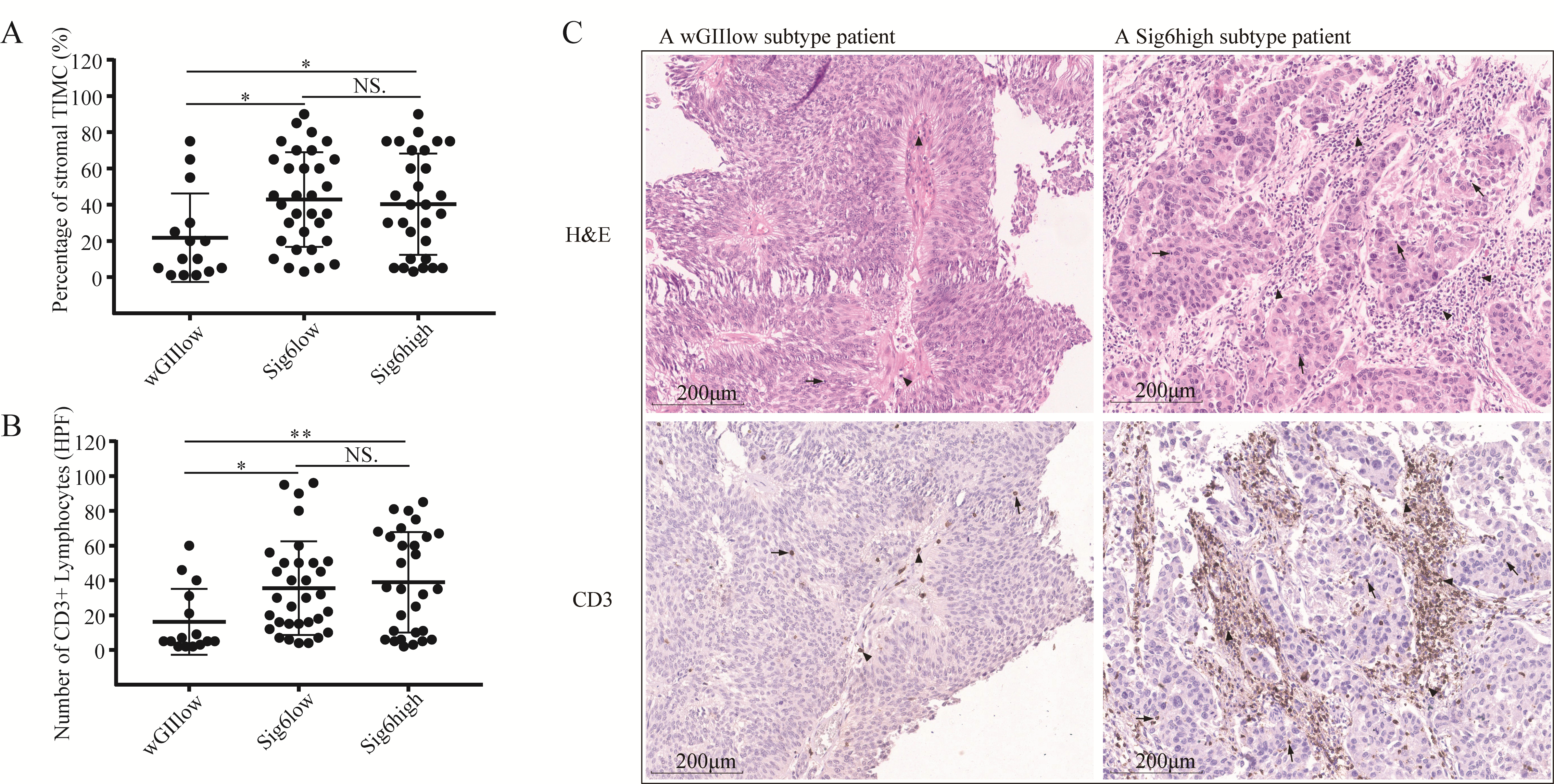


**Supplementary Figure 4.** Tumor-infiltrating lymphocytes in three subgroups. **(A)** The scatter plot showed the percentage of stromal TIMC stratified by three subgroups. **(B)** The scatter plot showed the number of CD3^+^ Lymphocytes stratified by three subgroups. Significant association between covariate and subtypes indicated by * *p* <0.05, ** *p* < 0.01 and *** *p* <0.001. **(C)** Tumor-infiltrating lymphocytes in Sig6high and wGII subgroups. Images of TIMCs and CD3^+^ lymphocytes of a representative patient from the Sig6high subgroup (right) and the wGIIlow subgroup (left). Triangle highlighted the TIMCs or CD3^+^ lymphocytes in stromal tumor region. Arrow highlighted the TIMCs or CD3^+^ lymphocytes in intra-tumor region. H&E = hematoxylin - eosin staining; TIMC = tumor-infiltrating mononuclear cell.


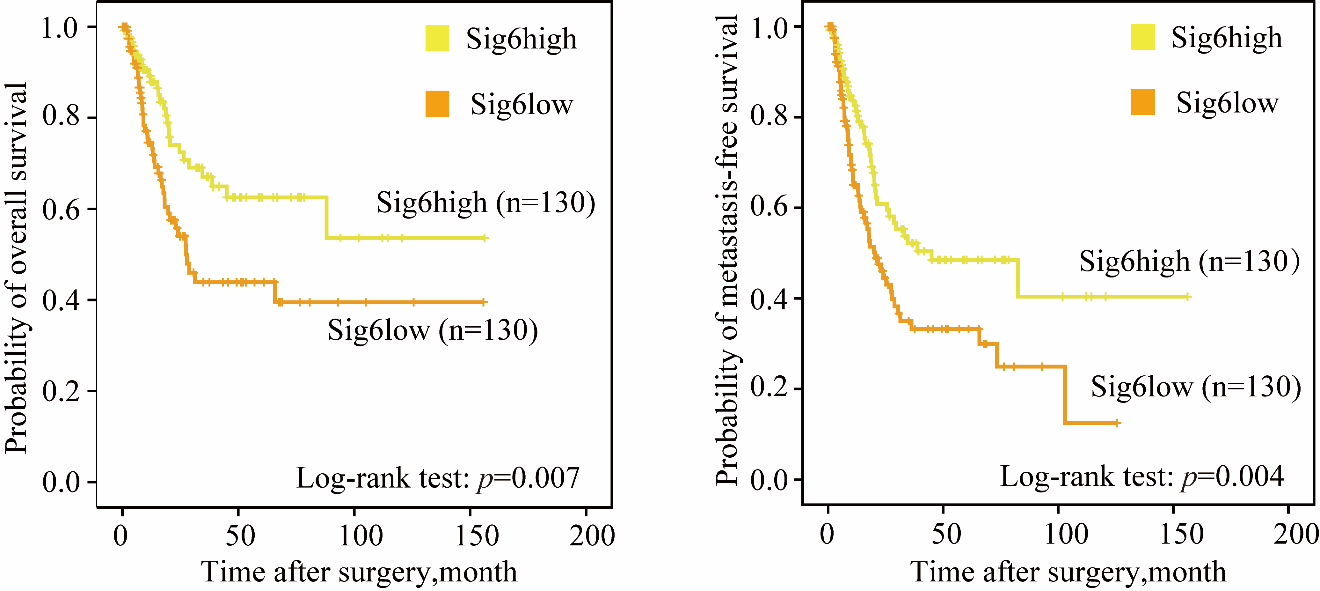


**Supplementary Figure 5.** Kaplan-Meier plots showed the difference of overall survival **(left)** and metastasis-free survival **(right)** in the UCB cohort from TCGA bisected by the spearman correlation with Sig6 around the median correlation.


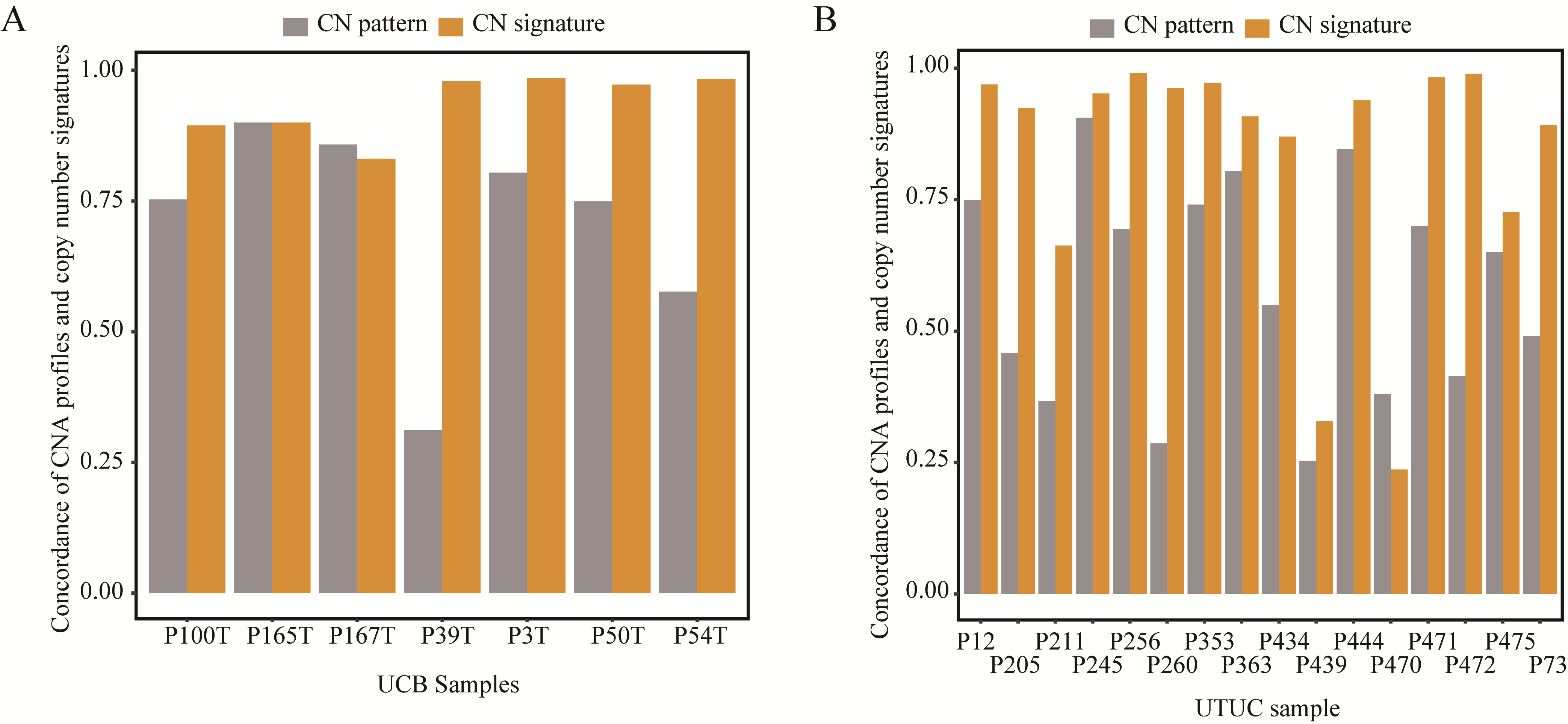


**Supplementary Figure 6.** Spearman correlation of CN profiles and CN signatures between cfDNA and matched primary tumor in 7 UCB **(A)** and 16 UTUC patients **(B).**

**
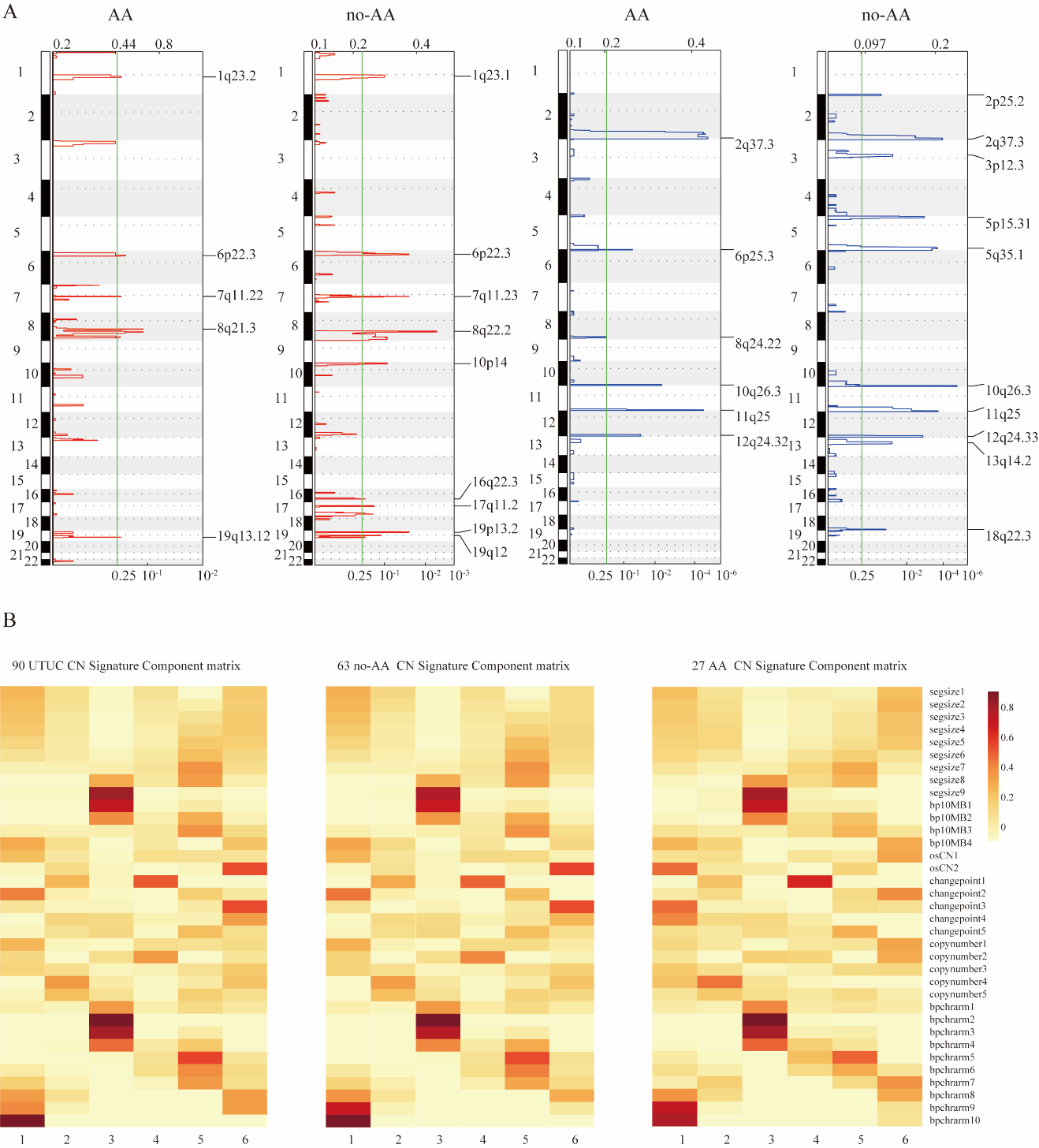
**

**Supplementary Figure 7.** Similar CN patterns and CN signatures were derived from AA and no-AA cohort of patient. **(a)** GISTIC plots show the comparison of significantly gained and loss regions between AA and no-AA patients (defined by SNV signature). **(b)** Heatmaps show comparison of component weights for CN signatures between 90 UTUC samples **(left)**, no-AA samples **(middle)**, and AA samples **(right)**.


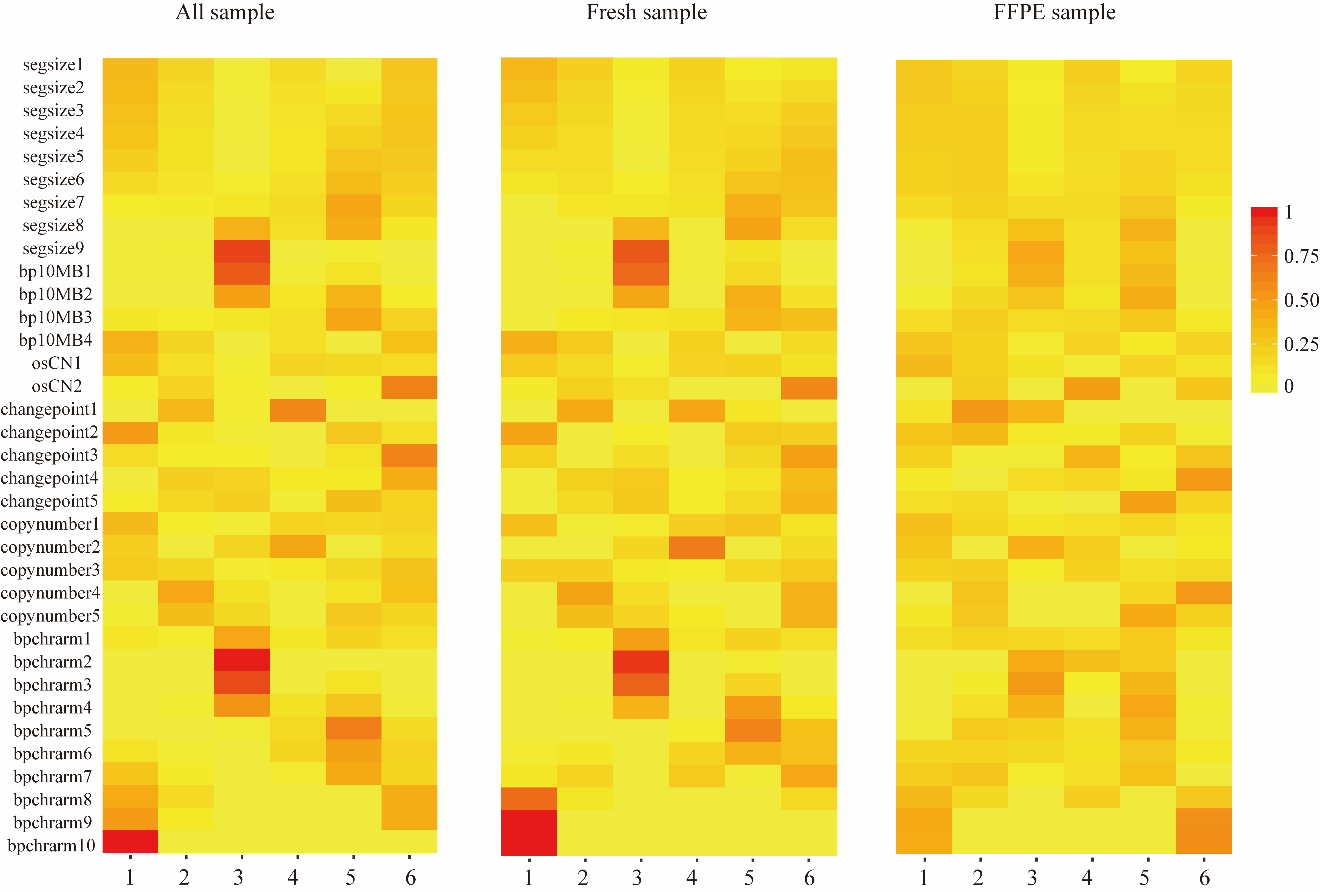


**Supplementary Figure 8.** Similar CN signatures were derived from fresh tumor tissue samples and formalin-fixed paraffin-embedded (FFPE) tumor samples. Heatmaps show component weights for CN signatures in 90 UTUC samples **(left)**, 43 fresh samples **(middle)** and 47 FFPE samples **(right)**.

**Supplementary Tables 1-5**

**Supplementary Table 1. The clinicopathologic variables of patients with collected preoperative urines.**

| **ID** | **Label** | **Age** | **Gender** | **Tumor stage** | **Grade** | **Paired tumor tissue** |
| --- | --- | --- | --- | --- | --- | --- |
| P195 | UCB | 60 | Male | T3a | 3 |  |
| P200 | UTUC | 58 | Male | T2 | 2 |  |
| P343 | UCB | 61 | Female | T1 | 2 |  |
| P346 | UCB | 63 | Male | T1 | 3 |  |
| P279 | UCB | 66 | Male | T1 | 3 |  |
| P245 | UTUC | 81 | Male | T2 | 3 | YES |
| P295 | UCB | 72 | Male | T2 | 3 |  |
| P363 | UTUC | 73 | Female | T2 | 2 | YES |
| P201 | UCB | 85 | Female | T1 | 3 |  |
| P229 | UCB | 70 | Male | T1 | 2 |  |
| P258 | UCB | 63 | Male | T1 | 2 |  |
| P254 | UCB | 63 | Male | T1 | 3 |  |
| P267 | UCB | 72 | Male | T4a | 3 |  |
| P208 | UCB | 71 | Male | T1 | 3 |  |
| P243 | UCB | 85 | Male | T1 | 3 |  |
| P259 | UCB | 67 | Male | T1 | 2 |  |
| P276 | UCB | 53 | Male | T1 | 2 |  |
| P227 | UCB | 76 | Male | T1 | 2 |  |
| P251 | UCB | 58 | Male | T1 | 2 |  |
| P204 | UCB | 85 | Male | T1 | 2 |  |
| P167 | UCB | 56 | Male | T2b | 3 | YES |
| P235 | UCB | 81 | Male | T3a | 3 |  |
| P100 | UCB | 63 | Female | T2b | 3 | YES |
| P12 | UTUC | 75 | Female | T2 | 2 | YES |
| P44 | UCB | 58 | Male | T1 | 2 |  |
| P54 | UCB | 71 | Female | T1 | 2 | YES |
| P108 | UCB | 79 | Male | T1 | 2 |  |
| P3 | UCB | 47 | Male | T1 | 2 | YES |
| P158 | UCB | 86 | Male | T1 | 2 |  |
| P50 | UCB | 87 | Male | T1 | 3 | YES |
| P165 | UCB | 50 | Male | T2b | 3 | YES |
| P39 | UCB | 60 | Male | T4a | 3 | YES |
| P17 | UCB | 49 | Male | T1 | 2 |  |
| P120 | UCB | 73 | Male | T1 | 2 |  |
| P130 | UCB | 72 | Male | T1 | 2 |  |
| P154 | UCB | 73 | Male | T1 | 2 |  |
| P202 | UCB | 78 | Male | Ta | 2 |  |
| P_2319215 | UCB | 65 | Male | T1a | 2 |  |
| p18 | UCB | 69 | Male | Ta | 2 |  |
| P53 | UCB | 64 | Male | T1 | 2 |  |
| P72 | UCB | 71 | Male | T2 | 3 |  |
| P74 | UCB | 55 | Male | T1 | 2 |  |
| P89 | UCB | 70 | Male | T1 | 1 |  |
| P91 | UCB | 65 | Male | T1 | 2 |  |
| P139 | UCB | 77 | Female | T1 | 3 |  |
| P143 | UCB | 49 | Male | T1 | 2 |  |
| P188 | UCB | 78 | Male | T4a | 3 |  |
| P219 | UCB | 71 | Male | Ta | 2 |  |
| P231 | UCB | 38 | Male | Ta | 1 |  |
| P236 | UCB | 68 | Male | T1 | 2 |  |
| P252 | UCB | 59 | Male | T1 | 2 |  |
| P253 | UCB | 68 | Male | Ta | 2 |  |
| P274 | UCB | 79 | Male | Ta | 2 |  |
| P348 | UCB | 77 | Male | T1 | 2 |  |
| P256 | UTUC | 66 | Male | T1 | 2 | YES |
| P16 | UTUC | 49 | Male | T1b | 2 |  |
| P298 | UTUC | 72 | Male | T3 | 3 |  |
| P333 | UTUC | 68 | Male | T3 | 3 |  |
| P353 | UTUC | 59 | Female | T3 | 3 | YES |
| P73 | UTUC | 69 | Male | T1 | 2 | YES |
| P80 | UTUC | 63 | Male | T1 | 3 |  |
| P205 | UTUC | 68 | Male | T1 | 2 | YES |
| P211 | UTUC | 66 | Female | T1 | 3 | YES |
| P215 | UTUC | 71 | Male | Ta | 2 |  |
| P260 | UTUC | 67 | Female | T1 | 2 | YES |
| P_2227545 | UTUC | 70 | Female | T2 | 2 |  |
| P431 | UCB | 71 | Male | T1 | 2 |  |
| P433 | UCB | 43 | Male | T1 | 1 |  |
| P434 | UTUC | 74 | Male | T2 | 3 | YES |
| P435 | UCB | 57 | Male | Ta | 2 |  |
| P437 | UTUC | 60 | Female | T3 | 3 |  |
| P439 | UTUC | 61 | Female | T1 | 2 | YES |
| P442 | UCB | 42 | Male | T1 | 3 |  |
| P443 | UCB | 50 | Male | T1 | 2 |  |
| P444 | UTUC | 68 | Male | T2 | 3 | YES |
| P445 | UCB | 63 | Male | T1 | 3 |  |
| P446 | UCB | 67 | Male | T1 | 2 |  |
| P447 | UCB | 76 | Male | T1 | 2 |  |
| P449 | UTUC | 46 | Male | T3 | 3 |  |
| P450 | UCB | 85 | Male | T1 | 1 |  |
| P451 | UCB | 77 | Male | T3b | 3 |  |
| P452 | UCB | 75 | Male | T2 | 3 |  |
| P467 | UTUC | 63 | Female | T1 | 3 |  |
| P468 | UCB | 62 | Male | T1 | 2 |  |
| P470 | UTUC | 54 | Female | T1 | 2 | YES |
| P471 | UTUC | 67 | Male | T1 | 2 | YES |
| P472 | UTUC | 57 | Male | T1 | 2 | YES |
| P475 | UTUC | 55 | Male | T3 | 2 | YES |
| P476 | UCB | 61 | Female | T1 | 2 |  |
| P477 | UCB | 79 | Female | T1 | 2 |  |
| P478 | UCB | 85 | Female | T1 | 2 |  |
| P481 | UCB | 55 | Male | T2a | 2 |  |
| P482 | UCB | 68 | Male | T1 | 2 |  |
| P483 | UCB | 60 | Female | T1 | 1 |  |

**Supplementary Table 2. Clinical characteristics of the Cohort I and Cohort II.**

| Variable | Cohort I  No. (%) | Cohort II  No. (%) | P-value |
| --- | --- | --- | --- |
| Total | 90 | 56 | - |
| Age  <65y  ≥65y | 40(44.4)  50(55.6) | 16(28.6)  40(71.4) | 0.055 |
| Smoking  Absent  Present | 74(82.2)  16(17.8) | 43(76.7)  13(23.2) | 0.423 |
| AA intake  Absent  Present | 63(70.0)  27(30.0) | 53(94.6)  3(5.4) | **<0.001** |
| Sex  Female  Male | 55(61.1)  35(38.9) | 33(58.9)  23(41.1) | 0.793 |
| CKD  1~2  3  4~5 | 38(42.2)  38(42.2)  14(15.6) | 24(42.9)  27(48.2)  5(8.9) | 0.485 |
| Primary tumour location  Pelvis  Ureter | 58(64.4)  32(35.6) | 27(48.2)  29(51.8) | 0.053 |
| Multifocality  Absent  Present | 78(86.7)  12(13.3) | 49(87.5)  7(12.5) | 0.884 |
| Tumour size  <3 cm  ≥3 cm | 38(42.2)  52(57.8) | 21(37.5)  35(62.5) | 0.571 |
| Architecture  Papillary  Sessile | 64(71.1)  26(28.9) | 38(67.9)  18(32.1) | 0.677 |
| T stage  Ta, 1  T2, T3&T4 | 43(47.8)  47(52.2) | 26(46.4)  30(53.6) | 0.874 |
| Grade  Low  High | 25(27.8)  65(72.2) | 24(42.9)  32(57.1) | 0.061 |
| N stage  N0 or Nx  N1~2 | 83(92.2)  7(7.8) | 51(91.1)  5(8.9) | 0.806 |

Nx: No lymph node dissection was performed.

**Supplementary Table 3. The correlation of CN signature exposure and CN signature subgroups**

|  | | **CN signature exposure** | | | | | | **Number** |
| --- | --- | --- | --- | --- | --- | --- | --- | --- |
|  | | **Sig1** | **Sig2** | **Sig3** | **Sig4** | **Sig5** | **Sig6** |  |
| **CN signature subgroups** | **wGIIlow (%)** | 3  (16.7) | 0  (0) | 3  (16.7) | 9  (50.0) | 3  (16.7) | 0  (0) | 18 |
|  | **Sig6low (%)** | 13  (36.1) | 12  (33.3) | 6  (16.7) | 4  (11.1) | 1  (2.8) | 0  (0) | 36 |
|  | **Sig6high (%)** | 12  (33.3) | 0  (0) | 0  (0) | 0  (0) | 3  (8.3) | 21  (58.3) | 36 |
| **Total** | | 28 (31.1) | 12 (13.3) | 9 (10.0) | 13 (14.4) | 7  (7.8) | 21 (23.3) | 90 |

CN = copy number.

**Supplementary Table 4. Univariate and multivariate Cox regression analysis predicting cancer-specific survival and metastasis-free survival for patients with no-AA mutational signature.**

| Variables | Cancer specific survival | | | | | | Metastasis-free survival | | | | | |
| --- | --- | --- | --- | --- | --- | --- | --- | --- | --- | --- | --- | --- |
|  | Univariable | | | Multivariable | | | Univariable | | | Multivariable | | |
|  | HR | 95% CI | *P* | HR | 95% CI | *P* | HR | 95% CI | *P* | HR | 95% CI | *P* |
| Age (<65 vs ≥65y) | 1.576 | 0.725~3.427 | 0.251 |  |  |  | 1.618 | 0.743~3.525 | 0.226 |  |  |  |
| Gender (Female vs Male) | 0.837 | 0.364~1.924 | 0.676 |  |  |  | 0.769 | 0.336~1.762 | 0.535 |  |  |  |
| Location (Ureter vs Pelvis) | 2.574 | 1.214~5.459 | **0.014** | 2.658 | 1.189~5.940 | **0.017** | 2.592 | 1.218~5.517 | **0.013** | 3.308 | 1.503~7.280 | **0.003** |
| Multifocality (Absent vs Present) | 0.597 | 0.180 | 1.980 | 0.399 |  |  | 0.623 | 0.188~2.067 | 0.439 |  |  |  |
| Tumor size (<3 cm vs ≥3 cm) | 0.847 | 0.402~1.782 | 0.662 |  |  |  | 0.810 | 0.385~1.705 | 0.580 |  |  |  |
| Architecture (Papillary vs Sessile) | 1.546 | 0.673~3.552 | 0.304 |  |  |  | 1.412 | 0.618~3.230 | 0.413 |  |  |  |
| T stage (T2, 3&T4 vs Ta, 1) | 4.884 | 2.264~10.534 | **<0.001** | 3.983 | 1.793~8.849 | **0.001** | 4.858 | 2.256~10.462 | **<0.001** | 4.552 | 2.062~10.046 | **<0.001** |
| Grade (Low vs High) | 2.580 | 0.895~7.442 | 0.079 |  |  |  | 2.629 | 0.911~7.584 | 0.074 |  |  |  |
| N (N1~2 vs N0/Nx) | 5.733 | 2.106~15.609 | **0.001** | 3.716 | 1.288~10.720 | **0.015** | 5.627 | 2.069~15.300 | **0.001** | 3.886 | 1.357~11.016 | **0.011** |
| CNV cluster (Sig6high vs Sig6low&wGIIlow) | 3.506 | 1.513~8.127 | **0.003** | 2.474 | 1.026~5.966 | **0.044** | 3.120 | 1.347~7.231 | **0.008** |  |  |  |
| MSI_ratio (≥0.15 vs <0.15) | 0.336 | 0.149~0.896 | **0.028** |  |  |  | 0.398 | 0.162~0.979 | **0.045** |  |  |  |

**Supplementary Table 5. Clinical characteristics of the subgroup patients stratified by CN signatures.**

| Variable | No. (%) | wGIIlow (%) | Sig6low (%) | Sig6high (%) | *P*-value |
| --- | --- | --- | --- | --- | --- |
| **Total** | 90 | 18 | 36 | 36 | - |
| **Age**  **<65y**  **≥65y** | 40(44.4)  50(55.6) | 8(44.4)  10(55.6) | 14(38.9)  22(61.1) | 18(50.0)  18(50.0) | 0.638 |
| **Smoking**  **Absent**  **Present** | 74(82.2)  16(17.8) | 14(77.8)  4(22.2) | 31(86.1)  5(13.9) | 29(80.6)  7(19.4) | 0.710 |
| **AA intake**  **Absent**  **Present** | 63(70.0)  27(30.0) | 16(88.9)  2(11.1) | 29(80.6)  7(19.4) | 18(50.0)  18(50.0) | **0.003** |
| **Sex**  **Female**  **Male** | 55(61.1)  35(38.9) | 10(55.6)  8(44.4) | 22(61.1)  14(38.9) | 23(63.9)  13(36.1) | 0.839 |
| **CKD**  **1~2**  **3**  **4~5** | 38(42.2)  38(42.2)  14(15.6) | 11(61.1)  6(33.3)  1(5.6) | 17(47.2)  15(41.7)  4(11.1) | 10(27.8)  17(47.2)  9(25.0) | 0.101 |
| **Primary tumour location**  **Pelvis**  **Ureter** | 58(64.4)  32(35.6) | 8(44.4)  10(55.6) | 24(66.7)  12(33.3) | 26(72.2)  10(27.8) | 0.124 |
| **Multifocality**  **Absent**  **Present** | 78(86.7)  12(13.3) | 17(94.4)  1(5.6) | 33(91.7)  3(8.3) | 28(77.8)  8(22.2) | 0.124 |
| **Tumour size**  **<3 cm**  **≥3 cm** | 38(42.2)  52(57.8) | 7(38.9)  11(61.1) | 17(47.2)  19(52.8) | 14(38.9)  22(61.1) | 0.735 |
| **Architecture**  **Papillary**  **Sessile** | 64(71.1)  26(28.9) | 10(55.6)  8(44.4) | 22(61.1)  14(38.9) | 32(88.9)  4(11.1) | **0.009** |
| **T stage**  **Ta, 1**  **T2, T3&T4** | 43(47.8)  47(52.2) | 10(55.6)  8(44.4) | 16(44.4)  20(55.6) | 17(47.2)  19(52.8) | 0.740 |
| **Grade**  **Low**  **High** | 25(27.8)  65(72.2) | 6(33.3)  12(66.7) | 10(27.8)  26(72.2) | 9(25.0)  27(75.0) | 0.812 |
| **N stage**  **N0 or Nx**  **N1~2** | 83(92.2)  7(7.8) | 13(72.2)  5(27.8) | 35(97.2)  1(2.8) | 35(97.2)  1(2.8) | **0.002** |
| **Adjuvant chemotherapy**  **Absent**  **Present** | 81 (90.0)  9 (10.0) | 15(83.3)  3(16.7) | 33(91.7)  3(8.3) | 33(91.7)  3(8.3) | 0.574 |
| **Adjuvant radiotherapy**  **Absent**  **Present** | 85 (94.4)  5 (5.6) | 16(88.9)  2(11.1) | 33(91.7)  3(8.3) | 36(100.0)  0(0.0) | 0.157 |

Nx: No lymph node dissection was performed.

**References:**

[1] Ge G, Peng D, Guan B, Zhou Y, Gong Y, Shi Y et al. Urothelial Carcinoma Detection Based on Copy Number Profiles of Urinary Cell-Free DNA by Shallow Whole-Genome Sequencing. Clin Chem 2020; 66(1):188-198.

[2] Lu H, Liang Y, Guan B, Shi Y, Gong Y, Li J et al. Aristolochic acid mutational signature defines the low-risk subtype in upper tract urothelial carcinoma. Theranostics 2020; 10(10):4323-4333.

[3] Endesfelder D, Burrell RA, Kanu N, McGranahan N, Howell M, Parker PJ et al. Chromosomal Instability Selects Gene Copy-Number Variants Encoding Core Regulators of Proliferation in ER+Breast Cancer. Cancer Research 2014; 74(17):4853-4863.

[4] Blokzijl F, Janssen R, van Boxtel R, Cuppen E. MutationalPatterns: comprehensive genome-wide analysis of mutational processes. Genome Med 2018; 10(1):33.

[5] Macintyre G, Goranova TE, De Silva D, Ennis D, Piskorz AM, Eldridge M et al. Copy number signatures and mutational processes in ovarian carcinoma. Nat Genet 2018; 50(9):1262-1270.

[6] Salipante SJ, Scroggins SM, Hampel HL, Turner EH, Pritchard CC. Microsatellite instability detection by next generation sequencing. Clin Chem 2014; 60(9):1192-1199.

[7] Niu B, Ye K, Zhang Q, Lu C, Xie M, McLellan MD et al. MSIsensor: microsatellite instability detection using paired tumor-normal sequence data. Bioinformatics 2014; 30(7):1015-1016.

[8] Song Y, Li L, Ou Y, Gao Z, Li E, Li X et al. Identification of genomic alterations in oesophageal squamous cell cancer. Nature 2014; 509(7498):91-95.

[9] Tate JG, Bamford S, Jubb HC, Sondka Z, Beare DM, Bindal N et al. COSMIC: the Catalogue Of Somatic Mutations In Cancer. Nucleic acids research 2018.

[10] Hendry S, Salgado R, Gevaert T, Russell PA, John T, Thapa B et al. Assessing Tumor-Infiltrating Lymphocytes in Solid Tumors: A Practical Review for Pathologists and Proposal for a Standardized Method from the International Immuno-Oncology Biomarkers Working Group: Part 2: TILs in Melanoma, Gastrointestinal Tract Carcinomas, Non-Small Cell Lung Carcinoma and Mesothelioma, Endometrial and Ovarian Carcinomas, Squamous Cell Carcinoma of the Head and Neck, Genitourinary Carcinomas, and Primary Brain Tumors. Advances in anatomic pathology 2017; 24(6):311-335.
